# The Nerve Supply to the Lumbar Region of the Dog With Special Reference to Cutaneous Innervation

**DOI:** 10.1111/ahe.70063

**Published:** 2025-09-15

**Authors:** Nicole Röhrmann, Christoph K. W. Mülling

**Affiliations:** ^1^ Institute of Veterinary Anatomy, Histology and Embryology, Faculty of Veterinary Medicine Leipzig University Leipzig Germany

**Keywords:** cutaneous innervation, dog, dorsal branch, spinal nerve, thoracolumbar region

## Abstract

Detailed anatomical information on the innervation of the skin by spinal nerve branches is fundamental for numerous integrative therapies, such as acupuncture or manual treatments. In recent years, interest in these therapies has grown, and they have gained increasing importance. Many musculoskeletal disorders in dogs are located in the region of the trunk, but knowledge about the course and branching of the cutaneous branches remains limited. This study presents a morphological analysis of the innervation pattern of the first cutaneous branch of the spinal dorsal nerve branches in 14 dogs. The dissection was performed in four layers to trace the cutaneous branches from the intervertebral foramen to their target area. The distances covered in each layer were measured and described using the Caudal Shift Index (CSI_*n*_). For this purpose, the ‘back region’ was defined as a dimensional unit, representing the distance between the spinous processes of two consecutive vertebrae within the examination area from the ninth thoracic (Th9) to the seventh lumbar (L7) vertebra. The results showed that the cutaneous branches innervating the lumbar region originated, on average, between Th11 and L4. The mean CSI_*n*_‐S was three back regions, indicating a distance equivalent to the length of three vertebral bodies between the nerve's origin at the foramen and the skin entry. In conclusion, these findings demonstrate consistency in the caudal shift of the cutaneous branches between Th9 and L4, with a high degree of symmetry between both sides of the body. These findings should be considered in therapeutic treatments.

## 1 Introduction

Morphological investigations in dogs and cats have shown that the assumption of similarity between the body sides and the manifestation of the branching pattern was not always confirmed (Bailey et al. 1984; Bernigau 2013; Bogduk 1976; Röhrmann et al. 2020).

Studies in cats have shown that cutaneous branches are not present at every dorsal spinal branch and are not always present bilaterally (Röhrmann et al. 2020). This has also been observed in studies on dogs (Bailey et al. 1984; Bernigau 2013; Haghighi et al. 1991). A dorsal branch divides into a medial branch for innervation of the muscles and a lateral branch, which supplies the skin. However, the lateral branch continues to the surface, where it divides into a lateral and medial cutaneous branch (International Committee on Veterinary Gross Anatomical Nomenclature 2017; Nickel et al. 2003). The branching into a lateral and medial branch can be seen regularly up to L3 in dogs; from L4 onwards, it is individual and no longer so regular (Frewein 1994). The medial cutaneous branch could not always be observed in dogs and cats (Bernigau 2013; Röhrmann 2017). Bogduk (1976, 1980, 1983) carried out studies in humans and cats to investigate and understand the possible causes of the back pain syndrome. This phenomenon is related to the theory of referred pain, highlighting the importance of understanding nerve supply.

Based on the known segmental anatomy of the spinal cord, specific tissue elements such as myotomes, sclerotomes, and dermatomes are segmentally associated. Blood vessels, like arteries and veins, also run segmentally to their corresponding dermatome. While macroscopic variability of the vessels is observed, histological examinations reveal a consistent pattern of the main vessels and a parallel arrangement of veins and arteries (Hughes 1960). It has been found that many nerve fibres travel into the hypodermis along with blood and lymph vessels (Palvetic 1991; Schwarz et al. 1979). This has been confirmed for many acupuncture points, which are located above vascular–nerve bundles that run between the body fascia (Egerbacher and Layroutz 1996; Heine 2016) and extend up to the body surface (Schaller 1956). These findings and their segmental relationship may explain the phenomenon of referred pain, commonly observed in musculoskeletal symptoms of the lower back. Consequently, therapies such as acupuncture, massage, neural therapy, and other manual treatments are used to alleviate the pain by introducing a new stimulus.

Interestingly, in cats, only the cutaneous branches of the spinal dorsal branch from the ninth thoracic (Th9) to the third lumbar (L3) vertebrae were consistently present and symmetrically detected (Röhrmann et al. 2020). Similar results were reported in dogs by Bailey et al. (1984), who observed that branches were consistently present up to L3 and partially present at L4 on both sides. In contrast, the investigation of Bernigau (2013) found cutaneous branches extending to L5 on one body side, with the highest innervation pattern in one dog exhibiting seven nerve branches between Th12 and L5. The most common finding was four nerve branches, observed in four of the dogs. These lumbar lateral cutaneous branches of the dorsal spinal branches are also referred to as cranial clunial nerves (Ellenberger et al. 1977; Frewein 1994). They innervate among others the skin of the gluteal region (Constantinescu 2018; Ellenberger et al. 1977; Frewein 1994; Nickel et al. 2003). Hermanson et al. (2019) describe that the cranial clunial nerves also originate from the branches of the medial or lateral branch of the dorsal branch of the caudal lumbar nerves. The middle clunial nerves are also cutaneous branches that originate from the sacral dorsal branches and supply the skin in the hip area, the caudal gluteal region as well as the sides of the upper thigh (Ellenberger et al. 1977; Frewein 1994; Nickel et al. 2003). However, the caudal clunial nerves originate from the ventral branches and are described as special branches of the sacral plexus or derivatives of the caudal femoral cutaneous nerve (Ellenberger et al. 1977; Frewein 1994; Henning 1965; Hummel 1965).

Additionally, ventral branches were occasionally observed at the innervating level of the spinal dorsal branches in both dogs and cats. These branches originated between L3 and L5 in six dogs and between Th12 and L3 in four cats. Nevertheless, no cutaneous branches of the spinal ventral branches were found at L6 and L7. Notably, the occurrence of the ventral branches innervating the dorsal skin was irrespective of the number of dorsal branches present (Bernigau 2013; Röhrmann et al. 2020). Bailey et al. (1984) did not mention such intersections between cutaneous branches of the spinal dorsal and ventral branches, but they provided a more detailed description of the location of the innervation areas of the nerve branches, focusing particularly on the most caudal branches between L2 and L4 in relation to the iliac crest.

Moreover, the study of Bernigau (2013) found that one dog had an individual anastomosis between the dorsal branches of Th13, L1 and L2, running parallel to the vertebral column. In this study, another nine dogs exhibited branches that split into two while perforating the thoracolumbar fascia or shortly after passing through it. Furthermore, in two dogs, nerve branches crossed the median plane and entered the contralateral skin.

All of these cutaneous branches of the spinal dorsal branches showed a caudal direction and a varying pattern from their origin to their supply. To classify this caudal shift between the intervertebral foramina and their termination at the skin, the Caudal Shift Index (CSI_*n*_) was defined (Bernigau 2013). The most superficial CSI_*n*_‐S at the skin had a mean value of three back regions. The CSI_*n*_‐F of the thoracolumbar fascia averaged two back regions, while the CSI_*n*_‐M at the deepest layer of the epaxial muscles measured one back region in both cats and dogs (Bernigau 2013; Röhrmann et al. 2020). Röhrmann (2017) also described a paramedian shift of the cutaneous branches through the fascia and musculature, whereby the distances between the spinous processes and the nerve passages through the fascia were shorter than in the musculature in the lumbar region. As a result, the lumbar cutaneous nerves followed a caudodorsal course in the fascia compared to the musculature.

The course and direction of the spinal nerve origins are generally described as caudal in anatomical textbooks for dogs, with one exception in the region between Th13 and L2, where they have a transverse orientation (Nickel et al. 2003).

The aim of the present study was therefore to provide a detailed description of the nerve supply to the lumbar region of the dog, with particular reference to the pattern of innervation of the cutaneous nerves.

## 2 Materials and Methods

### 2.1 Dissection and Data Collection

In this study, 14 adult dogs (nine male, five female) of different breeds and sex, weighing between 8.70 and 39.00 kg, were dissected and evaluated macroscopically (Table 1). All dogs had been euthanised in compliance with national and European animal welfare regulations by their referring veterinarian. Euthanasia was performed based on medical indications or terminal illness that is, for reasons unrelated to this study. The animals were subsequently donated to the Institute of Veterinary Anatomy for educational and scientific purposes. The investigations were approved by the Ethics Committee of the Veterinary Faculty, Leipzig University (EK 06/2023).

**TABLE 1:** Overview of the breed, sex and weight of the examined dogs.

	Breed	Sex	Weight (kg)
Dog 1	Mongrel, small	m	13.00
Dog 2	Mongrel, medium	m	39.00
Dog 3	Mongrel, medium	f	22.10
Dog 4	Mongrel, small	m	8.70
Dog 5	Mongrel, medium	m	18.20
Dog 6	Mongrel, medium	m	20.00
Dog 7	Mongrel, medium	m	20.00
Dog 8	German Sheperd	m	20.00
Dog 9	Labrador Retriever	m	30.00
Dog 10	Mongrel, medium	m	25.00
Dog 11	Mongrel, small	f	11.75
Dog 12	Rottweiler	f	38.00
Dog 13	Mongrel, medium	f	30.00
Dog 14	Irish Setter	f	25.00

Abbreviations: f, female; m, male.

The dissection was carried out on both sides of the body in four layers, focusing on the region between the ninth thoracic (Th9) and seventh lumbar (L7) vertebrae, as described by Röhrmann et al. (2020). This allowed for a comparison of body sides and a correlation with the published findings in cats. Unlike previous studies, the dogs in this investigation were non‐embalmed and dissected immediately upon arrival at the institute or after defrosting. For better visualisation, all lateral cutaneous branches of the spinal dorsal branches were marked in green, and the lumbar spinous processes were painted with acrylic paint—red (L1) and blue (L2 to L7).

To compare and analyse the course, branching, and innervation pattern of the lumbar dorsal branches in dogs with those in cats, the region between Th9 and L7 was selected.

**FIGURE 1:**
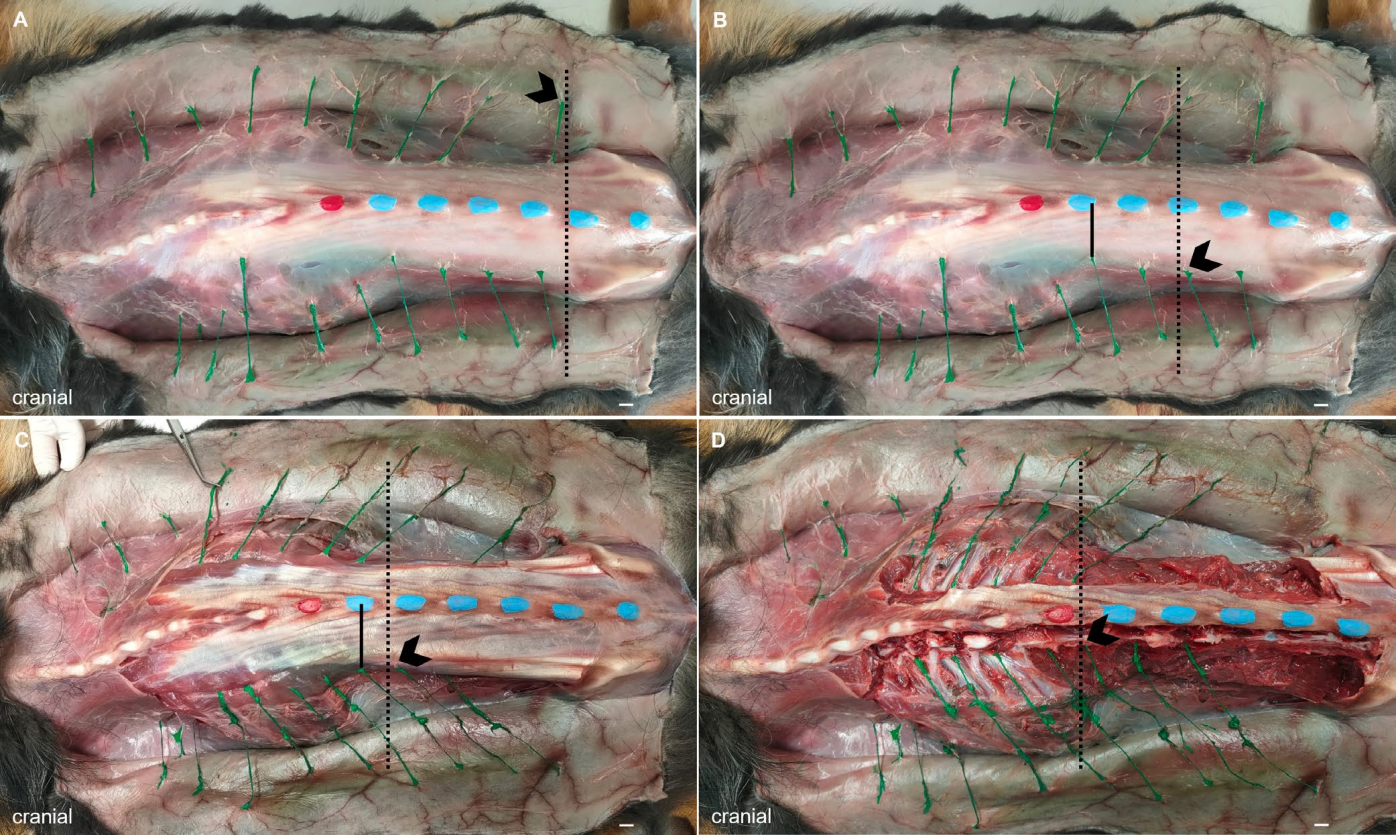
Illustration of the four dissected layers in dog 8. (A) First layer (skin), (B) second layer (fascia), (C) third layer (musculature), (D) fourth layer (intervertebral foramen). The dotted line and arrowhead indicate the point of entry (A) and the nerve passage (B–D) of the cutaneous nerve branches. The solid line indicates the distance between the spinous processes and the nerve passage (B, C). Scale bars: 1 cm.

The dissection started at the dorsal midline and was carried out in four layers (Figure 1). The first layer (layer 1—skin) was defined as the level at which the nerves enter the skin. The second layer (layer 2—fascia) was defined as the thoracolumbar fascia. The third layer (layer 3—musculature) was defined as the region above the epaxial muscles of the back, and the fourth layer (layer 4—intervertebral foramen) was defined as the level of the intervertebral foramina.

**FIGURE 2:**
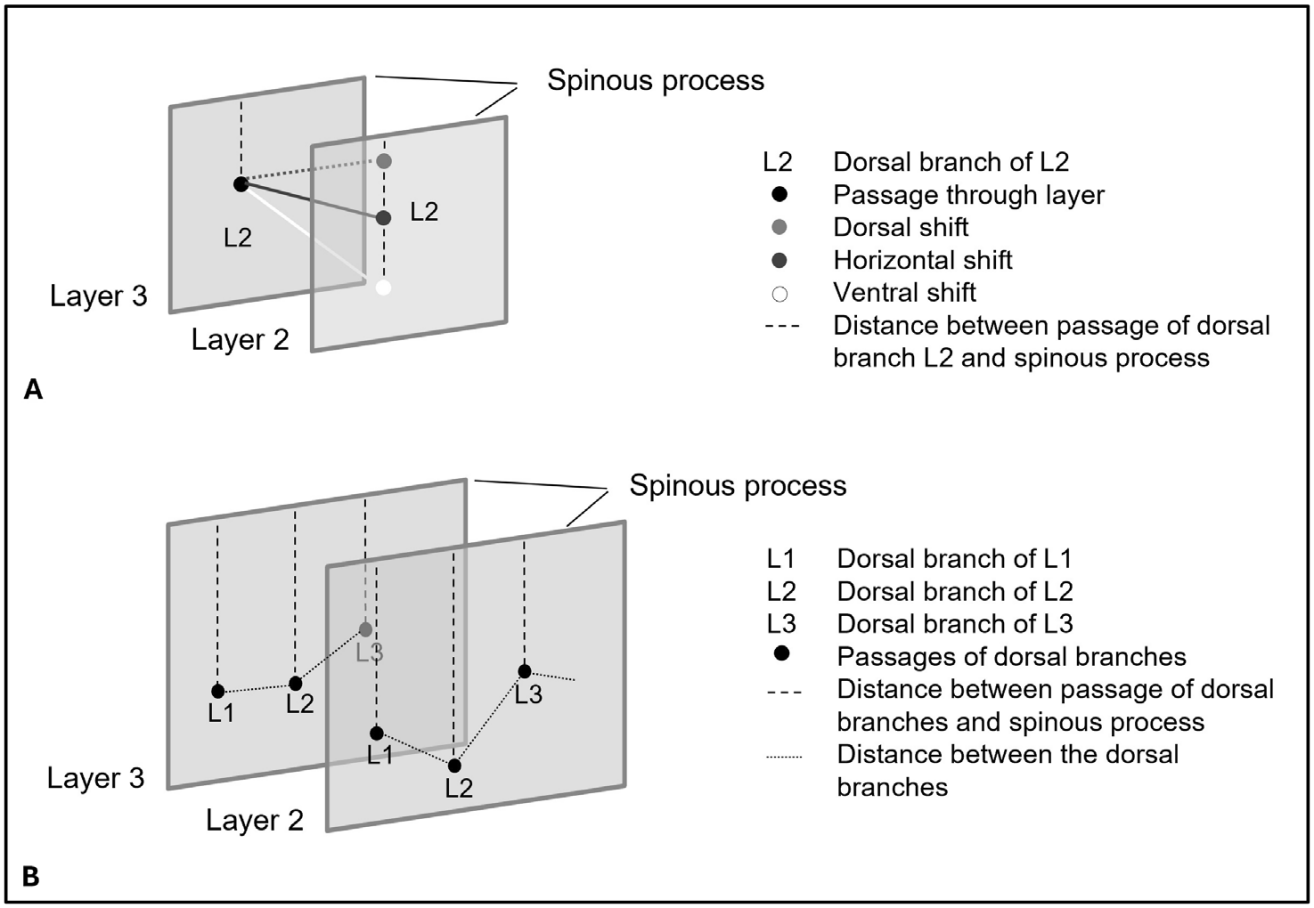
(A) Schematic representation of paramedian shift of dorsal branches between the spinous process and their passage in layers 2 and 3. (B) Illustration of the distance between the dorsal branches in each layer.

The cutaneous trunci muscle, along with fat and connective tissue, was removed while preserving the nerve branches to expose the thoracolumbar fascia (layer 2). Subsequently, the fascia and underlying tissue were removed to reach the level of the back muscles (layer 3). Finally, all epaxial muscles were carefully removed while protecting the nerve branches to identify the segmental origin of the nerves at the intervertebral foramina (layer 4). The course and branching pattern of the dorsal branches and their position relative to the vertebral bodies were documented and photographed in all layers. Furthermore, the distances between the nerves in all layers were measured, as well as the paramedian distance between the nerve passage and the spinous processes in layers 2 and 3 (Figures 1B,C and 2).

### 2.2 Determination of the Caudal Shift Index of the Nerves (CSI_*n*_ )

To compare the localization of nerve branches across different layers in relation to their origin in layer 4, a ‘back region’ was defined as the distance between the cranial borders of two consecutive spinous processes. This measurement was used to assess the Caudal Shift Indices (CSI_*n*_) as developed by Bernigau (2013). The CSI_*n*_ was calculated separately for layer 1 (skin, CSI_*n*_‐S), layer 2 (fascia, CSI_*n*_‐F), and layer 3 (musculature, CSI_*n*_‐M). The index was determined for each dorsal branch between Th9 and L7 on both sides of the body.

The Caudal Shift (*x*_*dn*_) of each nerve in every animal was calculated using the following formula, developed by Bernigau (2013):

CSI (*x*):

$$x_{\mathrm{dn}}=b_{\mathrm{dn}}-\bar{a_{\mathrm{dn}}}$$

*a*_*dn*_ = localization of the nerve branches in layer 1 (skin), layer 2 (fascia), or layer 3 (musculature) where the respective nerve branch (*n*) of a given dog (*d*) reaches the skin in the thoracolumbar region. The back region is defined by the distance between the cranial borders of two consecutive spinous processes. *b*_*dn*_ = localization of the nerve branches at the level of the transverse process in layer 4 (intervertebral foramen), where the respective nerve branch (*n*) of a given dog (*d*) originates. *d* = 1, …, 14. *n* = 1 (Th9), …, 12 (L7).

CSI for a particular nerve branch (CSI_*n*_), formula by Bernigau (2013):

$$\mathrm{CS}I_{n}=\frac{\sum_{d=1}^{d}x_{\mathrm{dn}}}{Z_{\mathrm{dn}}}$$

*z*_*dn*_ = number of dogs (*d*) possessing the respective nerve branch (*n*) in the specific thoracolumbar skin region. *d* = 1, …, 14. *n* = 1 (Th9), …, 12 (L7).

The ratio of the CSI_*n*_ in each layer, as well as the number of identified cutaneous branches of the spinal dorsal branches, was determined for all 14 dogs on both body sides. As described by Röhrmann et al. (2020), the mean CSI_*n*_ for a specific nerve was calculated by summing the CSI values and dividing by their number. This was performed for all nerve branches in the region between Th9 and L7 and for the individual layers (CSI_*n*_‐S, CSI_*n*_‐F, CSI_*n*_‐M). The CSI_*n*_‐S was used to describe the innervated skin areas of the canine thoracolumbar region.

### 2.3 Statistical Evaluation

To test for normal distribution, the Shapiro–Wilk test was applied, with significance set at *p* < 0.05. Subsequently, the Kruskal‐Wallis *H*‐test was used to compare CSI_*n*_‐S (layer 1—skin), CSI_*n*_F (layer 2—fascia) and CSI_*n*_‐M (layer 3—musculature) within each body side, with differences considered significant at *p* < 0.05. Significance between the paramedian distances of layers 2 (fascia) and 3 (musculature) for left and right was also determined using the Kruskal–Wallis *H*‐test. The Bonferroni correction was applied for post hoc correction, and the correlation coefficient was calculated according to Cohen (2013). To compare the layers as well as the paramedian distances in layer 2 (fascia) and 3 (musculature) between the body sides, the Mann–Whitney *U*‐test was used. The statistical evaluation was conducted using IBM SPSS Statistics (version 29.0.2.0 (20), 2023) and jamovi (version 2.7.4.0, 2025).

## 3 Results

### 3.1 Thoracic Cutaneous Branches

In eight dogs, all cutaneous branches from Th9 to Th13 were present on both sides. In one dog (dog 8), the Th9 branch was not visible at all. The branch of Th10 was found only on the right side in two dogs (dog 6 and 9) and only on the left side in one dog (dog 11). In two dogs (dog 3 and 11), the nerve branch of Th11 was detectable only on the left side, while in one animal (dog 12), it was present only on the right side. All 14 dogs had the branch of Th12 bilaterally. However, one dog (dog 11) had only a right branch of Th13 (Table 2).

**TABLE 2:** Presence of cutaneous branches of the dorsal branches from Th9 to L7 on the left, right or both sides of the body.

	Th9	Th10	Th11	Th12	Th13	L1	L2	L3	L4	L5	L6	L7
Dog 1	Both	Both	Both	Both	Both	Both	Both	Both	Both	—	—	—
Dog 2	Both	Both	Both	Both	Both	Both	Both	Both	Both	—	—	—
Dog 3	Both	Both	Left	Both	Both	Both	Both	Both	—	—	—	—
Dog 4	Both	Both	Both	Both	Both	Both	Left	Both	Both	—	—	—
Dog 5	Both	Both	Both	Both	Both	Both	Both	Both	Right	—	—	—
Dog 6	Both	Right	Both	Both	Both	Both	Both	Both	Both	—	—	—
Dog 7	Both	Both	Both	Both	Both	Both	Both	Both	Left	—	—	—
Dog 8	—	Both	Both	Both	Both	Both	Left	Both	—	—	—	—
Dog 9	Both	Right	Both	Both	Both	Both	Left	Both	—	—	—	—
Dog 10	Both	Both	Both	Both	Both	Both	Both	Both	Both	—	—	—
Dog 11	Both	Left	Left	Both	Right	Both	Both	Both	—	—	—	—
Dog 12	Both	Both	Right	Both	Both	Both	Both	Both	—	—	—	—
Dog 13	Both	Both	Both	Both	Both	Both	Both	Both	Left	—	—	—
Dog 14	Both	Both	Both	Both	Both	Both	Both	Both	—	—	—	—

### 3.2 Lumbar Cutaneous Branches

The L1 and L3 branches were identified bilaterally in all 14 dogs. Nonetheless, variations were observed in L2, where 11 dogs exhibited the branch bilaterally, while three dogs (dog 4, 8 and 9) had it only on the left side. For L4, the branch was found bilaterally in five dogs, only on the left side in two animals (dog 7 and 13), and only on the right side in one (dog 5). In six dogs (dog 3, 8, 9, 11, 12 and 14), the L4 branch was absent.

The cutaneous branches of the dorsal branches from L5 to L7 could not be traced in any of the 14 dogs (Table 2).

Ventral branches were not visible at the dissected level of the dorsal branches in any of the dogs examined.

### 3.3 Paramedian Shift and Branching Pattern (Nerve Courses)

To describe the branching pattern and course of the cutaneous branches of the spinal dorsal branches (Th9 to L4), the Caudal Shift Index (CSI_*n*_) was used. For the most superficial layer (CSI_*n*_‐S), values ranged from 1.82 ± 0.57 to 3.00 ± 0.84 on the left side and 1.92 ± 1.20 to 3.07 ± 1.16 on the right side. The overall mean was 2.58 (left) and 2.76 (right), approximately three back regions.

For the fascia layer (CSI_*n*_‐F), the mean shift was approximately two back regions, with an average of 1.99 (left) and 2.13 (right). The range extended individually to two and a half back regions, with values between 1.57 ± 0.49 and 2.57 ± 0.49 on the left and 1.83 ± 0.37 and 2.57 ± 0.62 on the right.

The deepest layer at the level of the back muscles had a mean CSI_*n*_‐M of one back region with 0.9 on the left and 1.00 on the right side. The CSI_*n*_‐M ranged from 0.46 ± 0.92 to 1.29 ± 0.58 on the left and from 0.31 ± 0.99 to 1.29 ± 0.58 on the right (Table 3).

**TABLE 3:** Mean values and standard deviations of the CSI_*n*_ for the nerve branches from Th9 to L7 in the skin (CSI_*n*_‐S), fascia (CSI_*n*_‐F), and musculature (CSI_*n*_‐M) on the left and right sides of the body.

	Left				Right			
	*n*	CSI_*n*_‐S	CSI_*n*_‐F	CSI_*n*_‐M	*n*	CSI_*n*_‐S	CSI_*n*_‐F	CSI_*n*_‐M
Th9	13	1.92 ± 1.43	1.69 ± 0.91	0.46 ± 0.92	13	1.92 ± 1.20	1.92 ± 0.82	0.31 ± 0.99
Th10	11	1.82 ± 0.57	1.82 ± 0.38	0.64 ± 0.64	14	2.36 ± 1.04	2.21 ± 0.67	0.64 ± 0.81
Th11	12	2.08 ± 0.49	2.42 ± 0.64	1.00 ± 0.00	13	2.85 ± 0.94	2.46 ± 0.63	1.00 ± 0.55
Th12	14	3.00 ± 0.84	2.57 ± 0.49	1.14 ± 0.63	14	3.07 ± 0.96	2.57 ± 0.62	1.14 ± 0.63
Th13	14	2.93 ± 0.79	2.29 ± 0.45	1.29 ± 0.58	13	2.85 ± 0.66	2.15 ± 0.53	1.15 ± 0.53
L1	14	2.93 ± 0.70	2.00 ± 0.37	1.14 ± 0.34	14	2.93 ± 0.79	2.14 ± 0.51	1.29 ± 0.58
L2	14	2.86 ± 0.63	1.86 ± 0.34	1.07 ± 0.45	12	2.92 ± 0.75	2.00 ± 0.57	1.17 ± 0.37
L3	13	3.00 ± 0.78	1.69 ± 0.46	1.08 ± 0.26	14	3.07 ± 1.16	1.86 ± 0.51	1.14 ± 0.34
L4	7	2.71 ± 1.03	1.57 ± 0.49	1.00 ± 0.00	6	2.83 ± 0.68	1.83 ± 0.37	1.17 ± 0.37
L5	0				0			
L6	0				0			
L7	0				0			

Most of the cutaneous branches showed a caudal direction, which was more pronounced on the surface compared to the intervertebral foramen and the musculature. However, in two dogs (dog 12 and 13), the right branch of Th9 showed a slight cranial direction. One dog (dog 14) displayed an exceptionally large shift, with a distance of up to 6 back regions between the fascia (layer 2) and the skin (layer 1).

**FIGURE 3:**
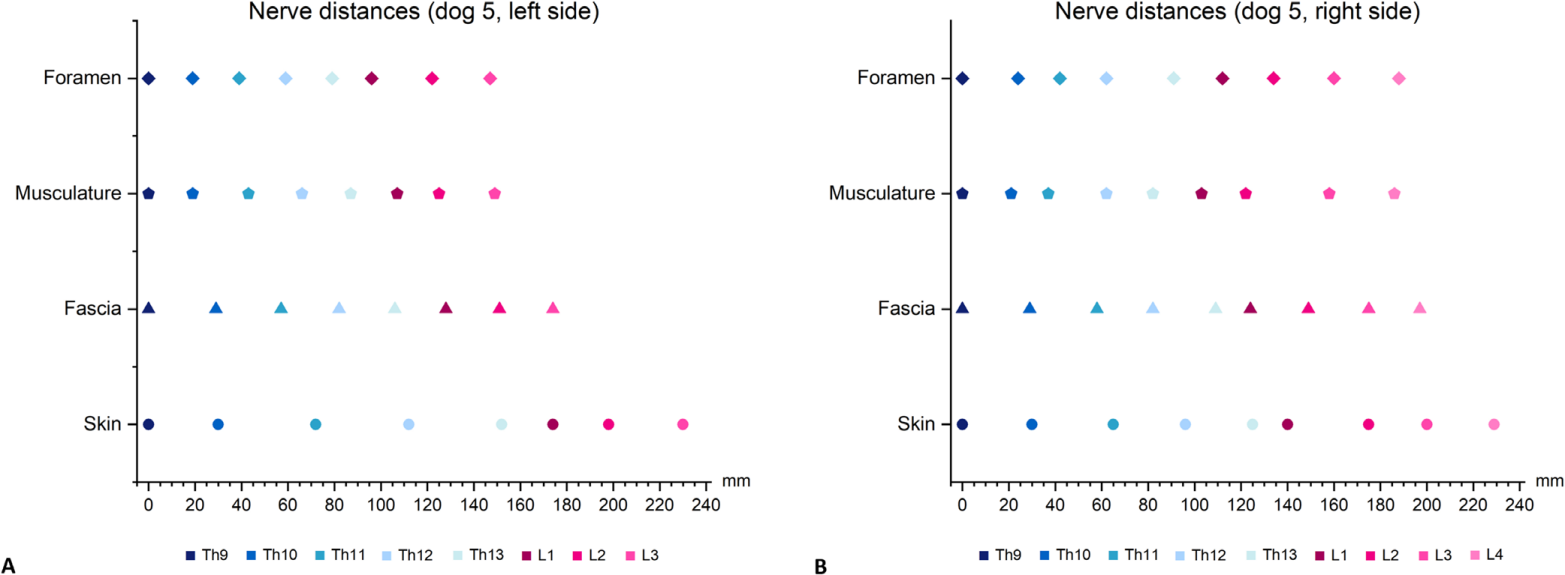
Distances between the nerves on either side of the body in all four layers. The intervals are plotted on the *x*‐axis (mm). The four layers are plotted on the *y*‐axis. (A) Distances between the cutaneous branches of dorsal branches Th9 and L3 on the left side. (B) Distances between the cutaneous branches of dorsal branches Th9 and L4 on the right side.

The distance between the cutaneous nerves varied across the layers from the inner to the outer regions; however, a consistent increase in the spacing between nerves was observed. The greatest distance between nerves was found in the first layer, the skin (Figure 3, Table 4). Differences in the distances between the intervertebral foramina on both sides of the body are likely due to the presence of residual soft tissue, which may have displaced the nerves to varying degrees.

**TABLE 4:** Summary of the distances (in mm) between the cutaneous branches of the dorsal branches Th9 to L4 in layer 1 (skin), layer 2 (fascia), layer 3 (musculature) and layer 4 (intervertebral foramen) on both sides.

Animal	Layer	Left									Right								
		Cutaneous branch									Cutaneous branch								
		Th9	Th10	Th11	Th12	Th13	L1	L2	L3	L4	Th9	Th10	Th11	Th12	Th13	L1	L2	L3	L4
Dog 1	4	0	21	20	19	20	20	18	24	24	0	20	20	20	20	18	24	24	26
	3	0	18	20	27	17	21	11	26	29	0	20	21	24	29	19	20	24	33
	2	0	21	31	33	20	14	14	18	23	0	30	34	32	21	13	31	10	19
	1	0	27	47	41	30	26	30	29	26	0	21	37	35	42	22	27	25	21
Dog 2	4	0	21	25	25	24	24	27	28	30	0	22	25	22	23	18	30	30	30
	3	0	21	22	30	26	17	26	25	27	0	23	29	24	24	25	27	32	25
	2	0	26	39	25	32	18	26	18	31	0	30	34	26	18	20	25	28	34
	1	0	31	44	32	39	45	22	14	30	0	40	40	30	24	23	35	30	28
Dog 3	4	0	25	28	28	28	28	32	35	—	0	21	—	35	24	28	34	34	—
	3	0	29	35	35	37	20	44	39	—	0	28	—	55	34	19	32	34	—
	2	0	30	35	37	30	29	30	33	—	0	32	—	56	34	26	30	28	—
	1	0	35	44	38	42	17	47	38	—	0	45	—	66	35	29	26	25	—
Dog 4	4	0	15	16	14	18	17	17	18	20	0	10	15	15	14	14	—	37	19
	3	0	14	17	22	15	18	15	20	25	0	21	12	22	24	14	—	16	20
	2	0	16	20	21	20	8	13	20	17	0	19	21	20	17	12	—	24	15
	1	0	22	20	20	21	15	35	18	16	0	29	21	25	18	24	—	25	11
Dog 5	4	0	19	20	20	20	17	26	25	—	0	24	18	20	19	21	22	26	28
	3	0	19	24	23	21	20	18	24	—	0	21	16	25	20	21	19	26	28
	2	0	29	28	25	24	22	23	23	—	0	29	29	24	27	15	25	26	22
	1	0	30	42	40	40	22	24	32	—	0	30	35	31	29	15	35	25	29
Dog 6	4	0	—	40	22	20	20	23	26	28	0	17	22	20	25	23	23	25	25
	3	0	—	42	20	40	24	25	35	17	0	20	20	23	25	25	20	30	25
	2	0	—	55	26	24	18	20	25	28	0	28	28	22	18	24	16	27	23
	1	0	—	58	37	30	32	26	31	35	0	25	30	36	30	30	20	35	35
Dog 7	4	0	18	18	25	24	26	25	32	34	0	23	27	26	26	25	26	37	—
	3	0	24	28	24	44	28	26	36	26	0	24	29	21	37	23	26	35	—
	2	0	30	42	26	31	20	28	25	30	0	38	32	30	27	21	21	30	—
	1	0	31	42	39	32	20	30	33	25	0	36	38	42	40	25	31	32	—
Dog 8	4	—	0	26	30	29	30	30	30	—	—	0	26	29	24	25		65	—
	3	—	0	22	31	26	27	28	37	—	—	0	29	31	33	22		79	—
	2	—	0	58	36	37	38	30	35	—	—	0	38	35	34	32		85	—
	1	—	0	65	42	30	35	39	24	—	—	0	45	32	64	35		46	—
Dog 9	4	0	—	44	26	25	27	26	31	—	0	21	24	25	27	26	23	29	—
	3	0	—	45	38	30	33	21	34	—	0	21	25	40	33	32	31	22	—
	2	0	—	27	48	39	30	20	26	—	0	30	37	28	35	30	25	29	—
	1	0	—	32	48	65	30	25	35	—	0	35	52	35	47	46	21	55	—
Dog 10	4	0	24	25	27	20	34	31	30	32	0	25	26	25	32	34	25	35	36
	3	0	24	35	26	32	25	45	28	23	0	29	31	33	42	37	38	31	22
	2	0	29	40	24	24	31	36	31	30	0	36	45	23	26	19	32	35	32
	1	0	39	37	38	37	35	34	45	30	0	39	42	35	25	29	43	41	32
Dog 11	4	0	—	—	29	16	16	21	15	—	0	15	15	15	—	30	13	14	—
	3	0	—	—	40	25	30	25	15	—	0	20	25	35	—	25	25	20	—
	2	0	—	—	55	26	40	14	12	—	0	18	18	22	—	28	28	25	—
	1	0	—	—	36	18	25	23	28	—	0	18	18	28	—	50	28	15	—
Dog 12	4	0	13	—	22	16	16	15	18	—	0	15	14	16	14	17	15	18	—
	3	0	35	—	40	46	30	25	43	—	0	30	45	30	37	32	30	40	—
	2	0	43	—	35	23	35	36	30	—	0	45	43	37	20	27	35	32	—
	1	0	35	—	65	45	74	55	45	—	0	60	55	46	33	23	50	25	—
Dog 13	4	0	15	11	23	17	13	25	25	27	0	15	20	13	11	20	26	20	—
	3	0	18	25	30	36	25	20	40	25	0	19	27	25	30	25	27	36	—
	2	0	27	30	27	26	20	20	24	37	0	32	28	40	30	20	28	23	—
	1	0	32	40	39	44	40	28	25	40	0	39	36	38	40	32	36	35	—
Dog 14	4	0	24	22	21	28	25	31	34	—	0	28	32	32	27	27	41	32	—
	3	0	25	31	24	20	35	34	34	—	0	26	35	25	20	25	32	34	—
	2	0	30	43	33	27	35	40	35	—	0	26	80	35	15	23	30	26	—
	1	0	35	35	30	22	25	30	35	—	0	54	85	50	50	40	33	75	—

*Note:* —, cutaneous branch was missing; 0, start of measurement in the examination area.

**FIGURE 4:**
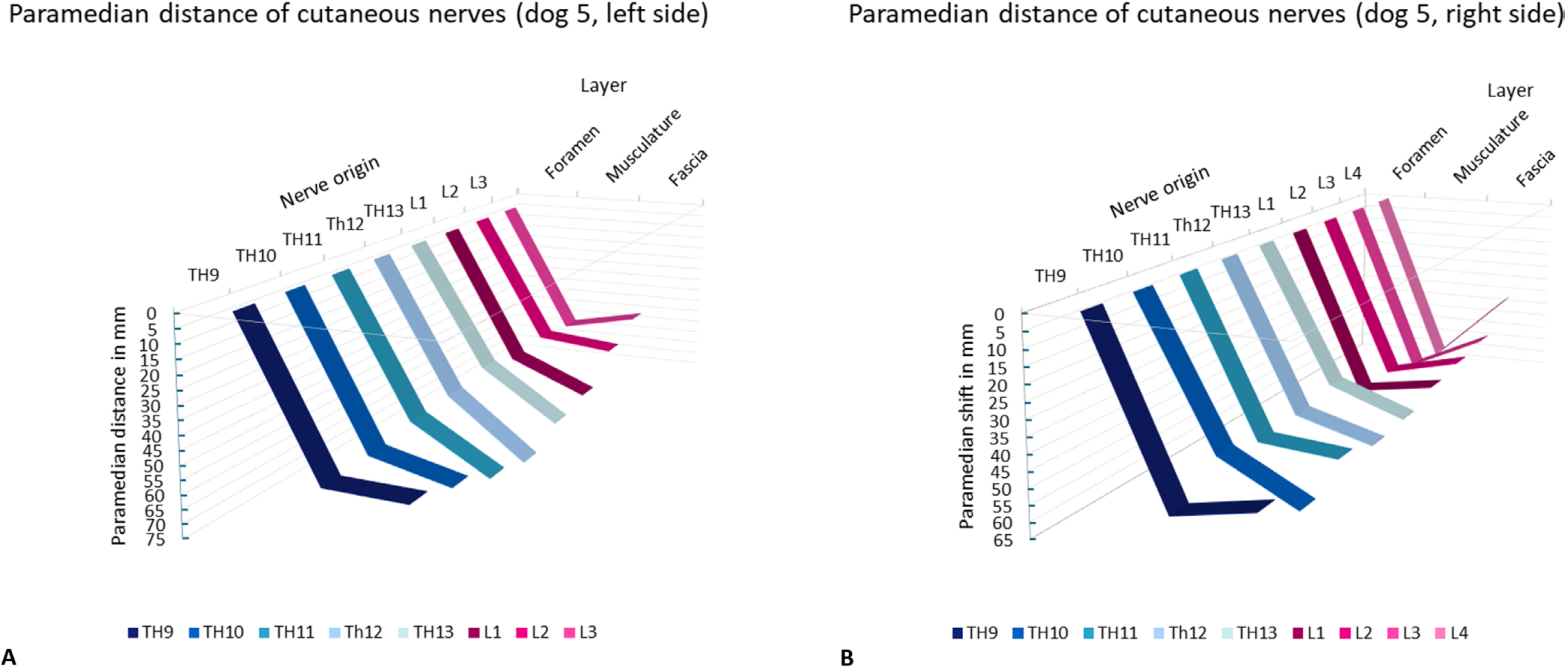
Paramedian distance of cutaneous nerves of dorsal branches Th9 to L4 in layer 2 and 3. (A) Paramedian shift (mm) of the branches Th9 to L3 on the left side of dog 5. (B) Paramedian shift (mm) of the branches Th9 to L4 on the right side of dog 5.

Comparison of the paramedian distances between layers 2 (fascia) and 3 (musculature) revealed that, in 13 out of 14 dogs on the left side and in 11 out of 14 dogs on the right side, the lumbar nerves followed a more caudodorsal course in layer 2 compared to layer 3 (Figure 4). Some nerves maintained a constant level when passing through the layers. This was observed for the left cutaneous branch of Th13 in dog 7, as well as for the right cutaneous branches of Th13 in dog 1, L1 in dogs 2, 6, and 8, and Th13 to L2 in dog 12 (Table 5).

**TABLE 5:** Overview of the paramedian passages (in mm) of the cutaneous branches of the dorsal branches Th9 to L4 through layer 3 (musculature) and layer 2 (fascia) of both body sides.

Animal	Layer	Left									Right								
		Cutaneous branch									Cutaneous branch								
		Th9	Th10	Th11	Th12	Th13	L1	L2	L3	L4	Th9	Th10	Th11	Th12	Th13	L1	L2	L3	L4
Dog 1	3	37	34	31	26	27	31	30	35	25	39	38	35	34	35	32	32	34	40
	2	57 ↓	53 ↓	48 ↓	48 ↓	44 ↓	40 ↓	43 ↓	41 ↓	33 ↓	68 ↓	49 ↓	47 ↓	40 ↓	35 =	33 ↓	42 ↓	35 ↓	29 ↑
Dog 2	3	64	62	66	59	52	55	62	62	65	64	62	58	58	60	65	69	75	79
	2	98 ↓	91 ↓	79 ↓	75 ↓	67 ↓	64 ↓	65 ↓	59 ↑	58 ↑	90 ↓	87 ↓	80 ↓	75 ↓	70 ↓	65 =	54 ↑	60 ↑	45 ↑
Dog 3	3	59	63	62	63	62	68	61	62	—	59	59	—	50	57	59	60	60	—
	2	78 ↓	78 ↓	80 ↓	73 ↓	68 ↓	64 ↑	56 ↑	50 ↑	—	92 ↓	87 ↓	—	69 ↓	65 ↓	61 ↓	52 ↑	49 ↑	—
Dog 4	3	38	38	37	39	37	36	42	40	39	34	35	34	30	30	33	—	34	39
	2	48 ↓	50 ↓	57 ↓	55 ↓	53 ↓	46 ↓	45 ↑	35 ↑	36 ↑	46 ↓	44 ↓	49 ↓	40 ↓	32 ↓	36 ↓	—	36 ↓	31 ↑
Dog 5	3	54	53	50	49	46	50	48	50	—	55	46	50	49	46	55	55	59	61
	2	67 ↓	71 ↓	65 ↓	62 ↓	51 ↓	44 ↑	39 ↑	39 ↑	—	59 ↓	52 ↓	56 ↓	55 ↓	51 ↓	49 ↑	47 ↑	37 ↑	35 ↑
Dog 6	3	50	—	44	40	44	48	50	43	45	50	50	46	50	46	50	52	57	42
	2	82 ↓	—	73 ↓	70 ↓	60 ↓	54 ↓	52 ↓	38 ↑	40 ↑	80 ↓	70 ↓	70 ↓	65 ↓	56 ↓	50 =	45 ↑	45 ↑	40 ↑
Dog 7	3	60	61	56	55	60	62	69	68	69	61	58	59	55	58	60	63	65	—
	2	87 ↓	85 ↓	70 ↓	66 ↓	60 =	55 ↑	47 ↑	46 ↑	44 ↑	80 ↓	75 ↓	75 ↓	63 ↓	54 ↑	51 ↑	50 ↑	48 ↑	—
Dog 8	3	—	54	55	62	62	68	66	81	—	—	53	51	57	54	55	—	52	—
	2	—	33 ↑	80 ↓	75 ↓	48 ↑	61 ↑	60 ↑	50 ↑	—	—	66 ↓	76 ↓	64 ↓	55 ↓	55 =	—	61 ↓	—
Dog 9	3	70	—	67	65	67	63	65	74	—	66	65	61	62	69	60	60	76	—
	2	95 ↓	—	98 ↓	85 ↓	75 ↓	70 ↓	68 ↓	65 ↑	—	90 ↓	95 ↓	91 ↓	87 ↓	76 ↓	74 ↓	65 ↓	65 ↑	—
Dog 10	3	59	55	60	59	58	64	62	64	64	60	59	54	55	53	52	51	52	56
	2	84 ↓	90 ↓	76 ↓	75 ↓	64 ↓	55 ↑	49 ↑	49 ↑	38 ↑	81 ↓	79 ↓	65 ↓	59 ↓	46 ↑	49 ↑	39 ↑	46 ↑	42 ↑
Dog 11	3	44	—	—	36	38	40	42	40	—	50	46	48	62	—	64	64	62	—
	2	60 ↓	—	—	50 ↓	50 ↓	38 ↑	48 ↓	50 ↓	—	70 ↓	60 ↓	60 ↓	46 ↑	—	60 ↑	60 ↑	54 ↑	—
Dog 12	3	54	60	54	64	66	50	46	40	—	36	38	50	48	46	42	42	38	—
	2	80 ↓	82 ↓	80 ↓	68 ↓	54 ↑	48 ↑	50 ↓	44 ↓	—	80 ↓	76 ↓	58 ↓	50 ↓	46 =	42 =	42 =	40 ↓	—
Dog 13	3	40	41	43	41	58	54	60	65	65	45	45	49	52	52	47	45	43	—
	2	65 ↓	61 ↓	58 ↓	51 ↓	68 ↓	47 ↑	48 ↑	53 ↑	53 ↑	70 ↓	65 ↓	64 ↓	62 ↓	62 ↓	40 ↑	38 ↑	36 ↑	—
Dog 14	3	61	56	54	60	68	77	77	79	—	65	62	73	59	59	62	50	61	—
	2	98 ↓	95 ↓	81 ↓	51 ↑	42 ↑	40↑	35 ↑	40 ↑	—	73 ↓	79 ↓	71 ↑	48 ↑	44 ↑	38 ↑	37 ↑	33 ↑	—

*Note:* —, cutaneous branch was missing; =, passage unchanged; ↑, passage more dorsal than in layer 3; ↓, passage more ventral than in layer 3.

### 3.4 Cutaneous Areas

**FIGURE 5:**
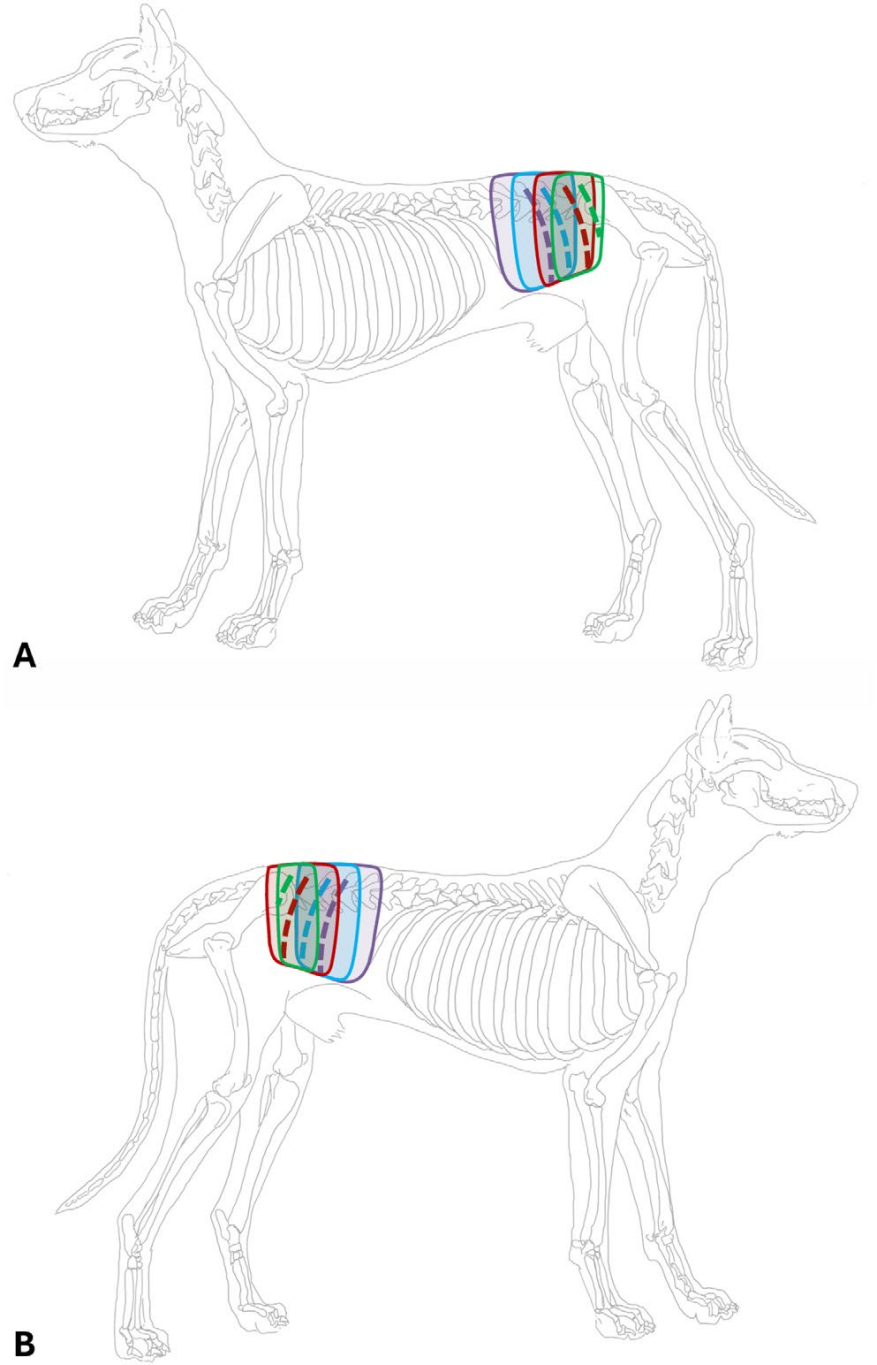
Representation of the nerve supply by the cutaneous branches of the dorsal spinal branches on the left (A) and right (B) side of the lumbar region. L1 (lilac), L2 (blue), L3 (red), L4 (green). The dotted line illustrates the mean CSI_*n*_‐S of the respective nerve branch.

The mean CSI_*n*_‐S values for the left and right cutaneous branches were nearly identical (Table 3). The minimum (CSI_*n*_‐S_min_) and maximum (CSI_*n*_‐S_max_) skin entry points were also similar (Table 6). For a better interpretation, CSI_*n*_‐S_min_, CSI_*n*_‐S_max_ and mean CSI_*n*_‐S are illustrated in Figure 5 to visualise the innervated lumbar cutaneous areas from L1 to L4. According to Röhrmann et al. (2020), these regions do not correspond to dermatomes, as only the subcutaneous entry point was considered. The figure shows that branches originating from L4 provide innervation extending to the sacral region (Figure 5).

**TABLE 6:** Comparison of dermatome borders identified by Bailey et al. (1984) with the CSI_*n*_‐S_max_ and CSI_*n*_‐S_min_ values from this study.

	Th9	Th10	Th11	Th12	Th13	L1	L2	L3	L4
Cranial border									
Bailey et al. 1984	Th_v_ 11–12	Th_v_ 13–L_v_ 1	L_v_ 1–2	L_v_ 2–3	L_v_ 3–4	L_v_ 4–5	L_v_ 5–6	L_v_ 6–7	L_v_ 7–S_v_ 1
CSI_*n*_‐S min left	Th_v_ 9	Th_v_ 11	Th_v_ 12	L_v_ 1	L_v_ 2	L_v_ 3	L_v_ 4	L_v_ 5	L_v_ 6
CSI_*n*_‐S min right	Th_v_ 9	Th_v_ 11	Th_v_ 13	L_v_ 1	L_v_ 2	L_v_ 3	L_v_ 4	L_v_ 5	L_v_ 6
Caudal border									
Bailey et al. 1984	Th_v_ 13–L_v_ 1	L_v_ 1–2	L_v_ 2–3	L_v_ 3–4	L_v_ 4–5	L_v_ 5–7	L_v_ 6–7	L_v_ 7–S_v_ 3	S_v_ 1–3
CSI_*n*_‐S max left	L_v_ 2	Th_v_ 13	L_v_ 1	L_v_ 4	L_v_ 5	L_v_ 6	L_v_ 6	S_v_ 1	S_v_ 2
CSI_*n*_‐S max right	L_v_ 1	L_v_ 2	L_v_ 3	L_v_ 4	L_v_ 4–5	L_v_ 6	L_v_ 6	S_v_ 2	S_v_ 1

### 3.5 Statistical Evaluation

The analysed data from both sides of the body does not follow a normal distribution for the CSI_*n*_. A comparison of the layer data between both body sides showed no significant differences with *p* = 0.799 for CSI_*n*_‐S, *p* = 0.478 for CSI_*n*_‐F, and *p* = 0.551 for CSI_*n*_‐M.

In contrast, the Kruskal–Wallis *H*‐test revealed significant differences within the layers for the left side (*χ* ^2^ = 8.707, df = 2, *p* = 0.013) and the right side (*χ* ^2^ = 9.072, df = 2, *p* = 0.009). Pairwise comparisons of the layers only showed a significant difference between CSI_*n*_‐S and CSI_*n*_‐M, with a strong positive correlation on both sides (left: *p* = 0.010, *r* = 0.60; right: *p* = 0.008, *r* = 0.61).

Normality testing of the distances between cutaneous nerves showed that, in most cases, the data followed a normal distribution. Exceptions to normality were detected for specific spinal levels in each layer: in layer 3 (musculature), Th9–Th10 and L2–L3 on the right side; in layer 2 (fascia), Th11–Th12 on the left side and Th10–Th11 as well as L2–L3 on the right side; and in layer 1 (skin), Th9–Th10 and Th13–L1 on the left side and Th11–Th12 as well as L2–L3 on the right side.

**FIGURE 6:**
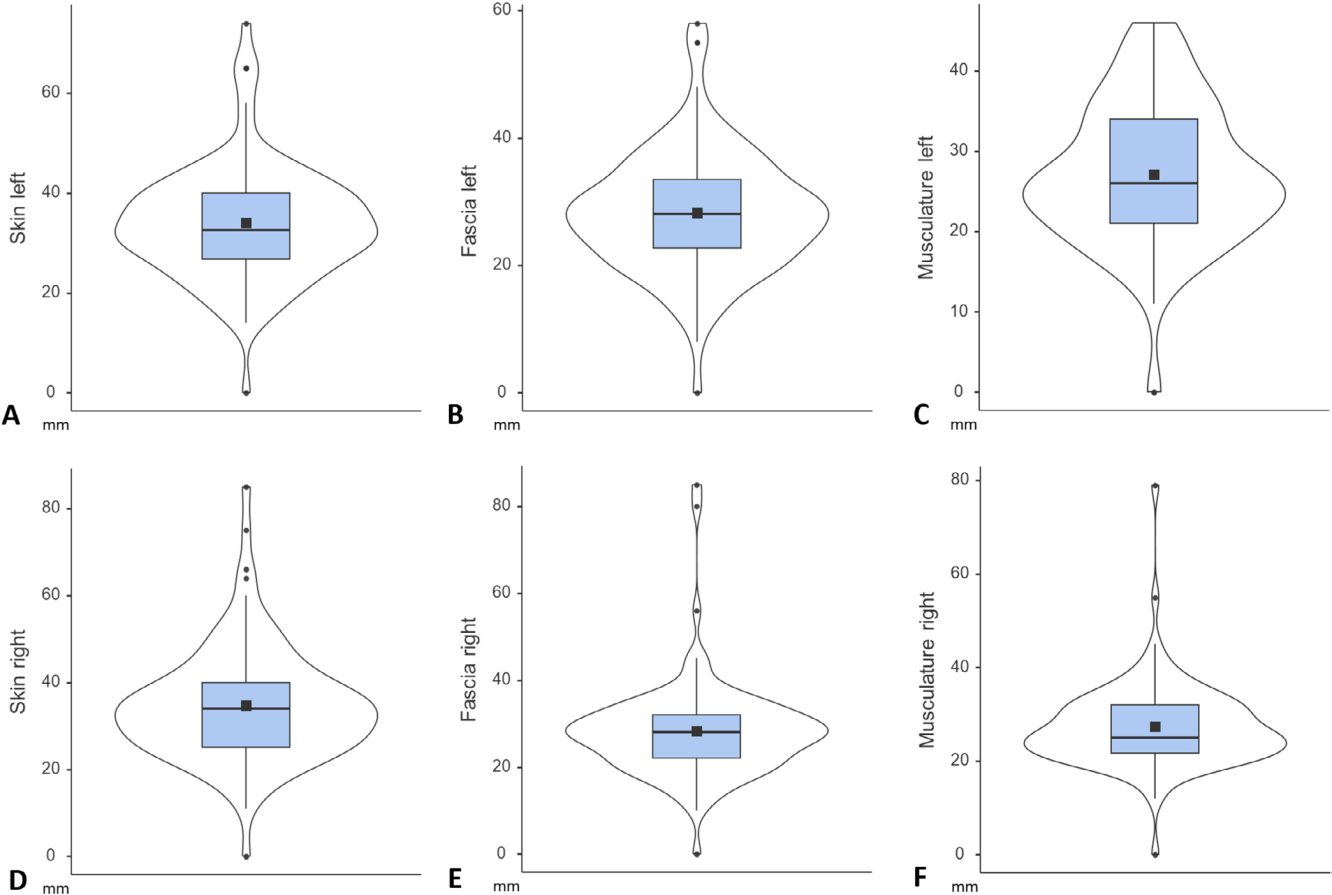
Violin plots illustrating the distribution of nerve distances (mm) across different tissue layers and body sides. Mean values are shown as squares and median values as horizontal lines inside the boxes. The box represents the interquartile range (IQR). The ends of the box indicate the lower 25th (Q1) and upper 75th (Q3) percentile. The whiskers reveal the minimum and maximum values and may appear asymmetrical due to variations in data distribution. Outliers are displayed as black dots outside the boxes. (A, D) Distances of cutaneous branches in layer 1 (skin). (B, E) Distances of cutaneous branches in layer 2 (fascia). (C, F) Distances of cutaneous branches in layer 3 (musculature).

The mean and median values in layer 2 (fascia) were the same on both sides, with a mean value of 28.2 mm and a median value of 28.0 mm. In layer 3 (musculature), the mean values of both sides differed only slightly, with 27.0 mm on the left and 27.3 mm on the right. The median values were 26.0 mm on the left and 25.0 mm on the right side. The mean values in layer 1 (skin) were 34.0 mm on the left and 34.6 mm on the right side, as well as the median values were 32.5 mm on the left and 34.0 mm on the right side (Figure 6).

The differences in paramedian distances between the layers were significant (left: *p* = 0.021; right: *p* = 0.046). However, the paramedian distances of the respective layers on both sides of the body showed no significant differences (layer 3 [musculature]: *p* = 0.171; layer 2 [fascia]: *p* = 0.258).

## 4 Discussion

In the 14 dogs examined, the number of dorsal branches innervating the thoracolumbar skin bilaterally ranged from five to nine cutaneous branches per side. These results differ from previous findings in those of cats (6 to 11) between Th9 and L7 (Röhrmann et al. 2020) and dogs (3 to 8) in the area of Th12 to L7 (Bernigau 2013).

During dissection, no cutaneous branches of the dorsal spinal branches L5, L6 and L7 were identified. The cutaneous branch of L3 was absent on the left side in one dog, and branches of L4 were irregularly present or completely absent, as described by Bailey et al. (1984) and Haghighi et al. (1991). In contrast, the findings in cats showed that the cutaneous branch of L6 was missing in all 14 cats, while the L7 branch was absent bilaterally in seven cats and unilaterally in four cats (Röhrmann et al. 2020). Bernigau (2013) found that cutaneous branches mostly occurred between Th12 to L3, with only a few branches originating from the segments L4 and L5. In addition, the cutaneous branches of L6 and L7 were absent in all 12 dogs studied. It cannot be completely ruled out that the nerve branches from L4 onwards were very fine and may have been severed or overlooked during dissection. However, the assumption of their absence or underdevelopment is supported by the statements of Hermanson et al. (2019), Frewein (1994) and Henning (1965), who describe that the cutaneous branches of the dorsal branches may occur inconsistently or be absent. For the animals examined here, it can be assumed that the irregularity in the development of the cutaneous nerves from L4 onwards is an individual difference between the animals. This seems to be confirmed by the data from Bernigau (2013), Bailey et al. (1984), and Haghighi et al. (1991). However, the absence of nerve branches from L5 onwards appears to occur regularly rather than being individual.

In the present study, neural anastomosis, contralateral course of the branches, or the distribution of the cutaneous branches of the dorsal branches at the fascia, as described by Bernigau (2013) were not observed. Furthermore, in contrast to the findings of Röhrmann et al. (2020) and Bernigau (2013), no cutaneous branches of the ventral branches were found at the innervation level of the dorsal branches.

All lumbar cutaneous branches exhibited a caudal shift on their path to the skin, a pattern previously observed in cats (Röhrmann et al. 2020). This finding is further supported by the analysis of the distances between the cutaneous branches, which were greatest in the first layer (skin). Consequently, both the mean and median values in this layer exceeded those observed in the other layers (Table 4, Figure 3). Bailey et al. (1984) also noted a progressive increase in caudal direction as nerves were located further caudal. However, in this study, no marked increase in nerve length could be confirmed. The mean CSI_*n*_‐S for the skin was consistently three back regions, aligning with findings in cats (Röhrmann et al. 2020). In this study, the mean values of the CSI_*n*_ were also about three back regions in layer 1 (CSI_*n*_‐S, skin), two back regions in layer 2 (CSI_*n*_‐F, fascia) and one back region in layer 3 (CSI_*n*_‐M, musculature). These results also correspond with previous findings in dogs of one body side (Bernigau 2013).

Statistical analysis further confirmed a significant and strong correlation between CSI_*n*_‐S and CSI_*n*_‐M, mirroring observations in cats. In this study, the distance between CSI_*n*_‐S and CSI_*n*_‐M is greatest and could thus explain the high correlation between the layers. However, unlike in cats, where a significant difference between CSI_*n*_‐F and CSI_*n*_‐M was detected, no such difference was observed in this study.

Anatomy textbooks describe the spinal nerves in dogs as following a caudal direction, except for the region between Th13 and L2. This description may be true for nerve fibres within the spinal canal. However, after leaving the intervertebral foramen, a consistent caudal shift was always observed throughout both the thoracic and lumbar regions. This caudal shift gradually increased from cranial to caudal. In addition, the lumbar cutaneous nerves usually showed a smaller paramedian distance in layer 2 (fascia) than in layer 3 (musculature), which overall indicates a more caudodorsal orientation of the course. This has also been described for cats (Röhrmann 2017). In summary, the lumbar cutaneous nerves predominantly follow a caudal course, with the greatest distances between them and a slight dorsal redirection along their path toward the skin.

Finally, the results of Bailey et al. (1984) regarding the cranial and caudal borders of the cutaneous areas of L2 to L4 align with the mean and maximum CSI_*n*_‐S values in this study, where the mean and maximum CSI_*n*_‐S of L2 were cranial to the iliac crest. The mean CSI_*n*_‐S of L3 was directly at the iliac crest and the mean CSI_*n*_‐S of L4 was slightly caudal to it; their respective maxima were located on the iliac crest. These findings partially correspond to dermatome descriptions by Bailey et al. (1984), particularly considering their statement that the cranio‐caudal extent of the cutaneous areas corresponds to approximately one and a half vertebral body lengths. The data also suggest an overlap of the innervated skin areas (Figure 5).

In conclusion, cats and dogs show marked differences in the presence of the caudal lumbar cutaneous branches. While both show an irregularity in the lumbar region, their thoracic branches are largely similar. The most striking difference is the presence of cutaneous branches caudal to L3. In dogs, no cutaneous branches were found beyond L4, whereas in cats, branches were occasionally present beyond L4. Despite the absence of lumbar cutaneous branches in the lower back, nerve supply to the skin is still present, as confirmed by the CSI_*n*_‐S values. This suggests that the cutaneous branches may follow an alternative route to reach the skin in the sacral region. Further research is needed to analyse the distribution pattern of the missing lumbar nerve branches.

## Conflicts of Interest

The authors declare no conflicts of interest.

## Data Availability

The data that support the findings of this study are available from the corresponding author upon reasonable request.
